# Health service pathways for patients with chronic leg ulcers: identifying effective pathways for facilitation of evidence based wound care

**DOI:** 10.1186/1472-6963-13-86

**Published:** 2013-03-08

**Authors:** Helen Edwards, Kathleen Finlayson, Mary Courtney, Nick Graves, Michelle Gibb, Christina Parker

**Affiliations:** 1 School of Nursing, Institute of Health and Biomedical Innovation, Queensland University of Nursing, Victoria Park Rd, Kelvin Grove, Australia; 2 School of Nursing, Institute of Health and Biomedical Innovation, Queensland University of Technology, 60 Musk Ave, Kelvin Grove, 4059, Australia; 3 School of Nursing, Midwifery and Paramedicine, Australian Catholic University, 1100 Nudgee Rd, Banyo, 4014, Australia

**Keywords:** Chronic leg ulcer, Health services, Evidence based practice

## Abstract

**Background:**

Chronic leg ulcers cause long term ill-health for older adults and the condition places a significant burden on health service resources. Although evidence on effective management of the condition is available, a significant evidence-practice gap is known to exist, with many suggested reasons e.g. multiple care providers, costs of care and treatments. This study aimed to identify effective health service pathways of care which facilitated evidence-based management of chronic leg ulcers.

**Methods:**

A sample of 70 patients presenting with a lower limb leg or foot ulcer at specialist wound clinics in Queensland, Australia were recruited for an observational study and survey. Retrospective data were collected on demographics, health, medical history, treatments, costs and health service pathways in the previous 12 months. Prospective data were collected on health service pathways, pain, functional ability, quality of life, treatments, wound healing and recurrence outcomes for 24 weeks from admission.

**Results:**

Retrospective data indicated that evidence based guidelines were poorly implemented prior to admission to the study, e.g. only 31% of participants with a lower limb ulcer had an ABPI or duplex assessment in the previous 12 months. On average, participants accessed care 2–3 times/week for 17 weeks from multiple health service providers in the twelve months before admission to the study clinics. Following admission to specialist wound clinics, participants accessed care on average once per week for 12 weeks from a smaller range of providers. The median ulcer duration on admission to the study was 22 weeks (range 2–728 weeks). Following admission to wound clinics, implementation of key indicators of evidence based care increased (p < 0.001) and Kaplan-Meier survival analysis found the median time to healing was 12 weeks (95% CI 9.3–14.7). Implementation of evidence based care was significantly related to improved healing outcomes (p < 0.001).

**Conclusions:**

This study highlights the complexities involved in accessing expertise and evidence based wound care for adults with chronic leg or foot ulcers. Results demonstrate that access to wound management expertise can promote streamlined health services and evidence based wound care, leading to efficient use of health resources and improved health.

## Background

Many adults with vascular disease and/or diabetes suffer with chronic leg or foot ulcers, leading to loss of functional ability, poor quality of life and long term ill-health [1]. Studies on patients with chronic leg ulcers have reported the average duration of these ulcers is around 12–13 months [2,3], around 60–70% of patients have recurring ulcers [4], 24% of patients are hospitalised because of the ulcers and most people suffer from the condition for an average of 15 or more years [3]. Care for chronic wounds is reported to cost 2-3% of total health care spending in developed countries [5,6] and these costs are set to rise with ageing populations [7]. Treatment in the U.S. costs over 3 billion $US and the loss of over 2 million workdays a year [6]. Similarly, Harding quotes a cost of £400 million each year in the U.K. [8]. In Australia, wound dressings are the second most frequent procedure in General Practitioner practice [9] and chronic wound care accounts for 22–50% of community nursing time in the UK and Australia [10,11]. In addition to direct health care costs, chronic wounds are associated with hidden burdens on the community resulting from loss of mobility, decreased functional ability, social isolation and loss of participation in the workforce and society.

Despite reports of improved healing and reduced recurrence rates following the introduction of evidence based guidelines and coordinated care [6,12], a significant evidence-practice gap has been reported around the world in appropriate assessment of chronic leg ulcers and timely use of best practice treatments [13-18]. For example, around 70% of chronic leg ulcers are caused by venous disease and compression therapy is the gold standard treatment [19], yet a U.S. study found only 17% of patients with venous leg ulcers received compression [14], and Australian studies found 40–60% of venous leg ulcers in Australia did not receive adequate compression [16,20].

A number of reasons have been identified as contributing to this evidence-practice gap, including lack of information and skills [15,17], difficulties with access to evidence based guidelines [14], the costs and lack of reimbursement associated with specialist wound care and treatments such as compression bandaging [14,21], limited access to specialist multidisciplinary teams [22], poor communication [15] and limited evidence on effective assessment, referral and treatment pathways of care to manage this chronic condition [15]. Coyer et al. [15] found clients were confused as to whom to access for care (whether general practitioners, community clinics, pharmacists, outpatient departments, vascular specialists, skin specialists); and health professionals themselves often find it difficult to manage care across disparate levels (community nurses, general practitioners, vascular/endocrine/wound care specialists, allied health professionals) in health care systems which lack models of service delivery that integrate chronic disease primary care and focus on health promotion, illness prevention and early intervention.

In the area of wound healing many practitioners are involved in the trajectory of care. The absence of wound care as a medical specialty and dispersion of responsibility for wound care among a variety of health care providers often results in poor continuity of care across the health service continuum and a lack of consistent, evidence-based care and long-term preventative care [23,24]. The diversity of budgets and financial climate of cost control means that there is extraordinary complexity in the funding and provision of wound care and preventive care in the community [14,25]. Up-front costs for long term wound care (wound dressings, bandages, costs of health care service providers) and follow-up preventative care have been identified as a barrier to implementing evidence based practice [14,15].

The potential benefits of specific health service pathways for chronic leg ulcer management and facilitation of evidence based wound care are not clear from current research. A few studies have demonstrated improved clinical outcomes following the introduction of evidence based protocols [26-28], however, the relative benefits (both in patient outcomes and effective use of health resources) of alternative models of care are not well evaluated. This area of translational research is important in addressing gaps between research findings and wide-spread implementation of new information to improve patient outcomes.

This project was conducted in Queensland, a state of Australia, which has complex and diverse systems of health care provision and funding, differing in each state. For the participants in this study in Queensland, reimbursement varies according to the health care providers. A base level rebate is provided by the government for visits to a General Practitioner or medical specialist (on referral), and some patients are charged this amount (i.e. no cost to the patient), while others are charged an additional fee each visit as determined by the General Practitioner or medical specialist. Upon referral from a medical practitioner, patients can access a consultation at outpatient specialist wound clinics at public hospitals at no cost.

Community nursing services are provided primarily by non-government not-for-profit organisations with government funding to support the cost for eligible patients i.e., those who are aged over 65 years (or over 50 years for Aboriginal and Torres Strait Islander patients), or disabled, or those who are at risk of premature or inappropriate admission to long term residential care. There is usually a top-up fee for the patient each visit in addition to the costs for consumables. Participants receiving allied health professional services (e.g. podiatrists, occupational therapists, physiotherapists) in the community would usually incur the full costs of consultations, although some may be eligible for some reimbursement of costs with a referral from a medical practitioner, or may have private health insurance to cover some of the costs. The costs associated with dressings and bandaging are not subsidised for community living patients attending any health or allied health service provider, and as these may be substantial, it often influences choice of treatments.

The aim of this project was to explore the effectiveness of alternative health service pathways of care for patients with chronic leg ulcers, on

•implementation of evidence-based guidelines;

•wound healing and recurrence rates; and

•efficient use of health services and cost-effectiveness of care.

## Methods

This project had two phases, a retrospective and prospective phase. The retrospective study utilised a survey and chart audit exploring existing health service pathways of management, referrals and outcomes during the twelve months prior to enrolment in the study. The prospective phase involved a longitudinal observational study of participants attending one of two specialist wound services for care of a chronic leg ulcer to determine outcomes over six months of care.

### Sample and site

All patients with a chronic leg ulcer and fitting the inclusion and exclusion criteria were invited to participate in the study. Patients were recruited from two study sites – one site was a community stand alone specialist wound clinic; the other site was an outpatient specialist wound clinic within a large tertiary metropolitan hospital. To gain access to the hospital outpatient wound clinic, patients are referred by their General Practitioner or other medical practitioner. A medical practitioner specialising in wound care runs the outpatient clinic with the assistance of nurses with wound care expertise. There is no charge (apart from travel and parking) for the patients. The community stand alone clinic is based within a university health clinics site, and accepts patient self-referral or referral from medical or other health service providers. The service is led by a Nurse Practitioner in Wound Management, assisted by nurses with expertise in wound care. There is normally a small charge for visits to the clinic, however, if patients are unable to pay, the charge is waived. Both study clinics had access to multidisciplinary health professional networks as appropriate. Patients agreeing to participate in the study at either site were offered free bandages for the duration of the study.

#### Inclusion criteria

Clients who presented with a leg or foot ulcer below the knee

#### Exclusion Criteria

Clients who were unable to speak or understand English

Clients who were cognitively impaired

Leg ulcers involving malignancy

### Data collection and measures

Information on demographics, medical history and variables known to influence healing rates were collected upon admission to the study, including age, income, socioeconomic status, general health, medical and venous history, comorbidities, previous leg ulcer/s history (time of first onset, number, time to healing, time to recurrence), current ulcer history and clinical assessment (size, duration, site, tissue type, Ankle Brachial Pressure Index and a neuropathic foot assessment).

In the retrospective phase, data on previous health service pathways during the twelve months prior to admission to the leg ulcer clinic (including referrals and wound management) were determined via participant surveys and interviews. Data were collected on cost effectiveness measures (type of health services provided, investigations, types of dressings and bandages used, occasions of care, allied health and/or community services required, loss of functional ability); and health service pathway information (current and previous treatments, investigations, wound dressings, bandage and/or compression types, health service providers, referrals, occasions of care, allied health and community services required). In the prospective phase, data on health service pathways and cost effectiveness measures (as above for Phase 1), wound management and treatments, and wound healing outcomes were measured weekly for 24 weeks from admission. In addition data on quality of life, pain and functional ability were collected on admission and then at 12 and 24 weeks.

Progress in wound healing was measured with the following methods:

wound tracings, digital planimetry and digital photography to determine i) ulcer area, ii) ulcer area reduction over time, iii) percentage area reduction and iv) healing rates (numbers totally healed);

the PUSH tool for ulcer healing [29], which takes into account type of exudate and wound bed tissue type (i.e. epithelial, granulating, slough or necrotic) and has been validated for use with chronic leg ulcers [30,31]; and

clinical data related to healing progress such as presence of oedema, eczema, inflammation, signs of infection.

Quality of life, pain, functional ability and psychosocial data were measured with the SF-12 [32], Medical Outcomes Study Pain Measures [33], Geriatric Depression Scale [34] and Instrumental Activities of Daily Living Scale [35].

### Analysis

Descriptive statistics were calculated for each of the baseline, primary and secondary outcome measures. T-tests, ANOVAs, Mann–Whitney U or Kruskal-Wallis tests were undertaken to identify relationships between health service providers and demographic or clinical independent variables. Repeated measures ANCOVAs were undertaken to analyse differences in pain, depression and health-related quality of life scale scores over time. Kaplan-Meier survival curves were calculated to determine median time to healing for participants in the prospective study.

#### Ethics

This study received ethical approval from the Human Research Ethics Committees at each of the participating organisations and complied with the Declaration of Helsinki ethical rules. Written informed consent was obtained from all participants.

## Results

A sample of 104 participants was recruited for the retrospective study and 70 of this group also participated in the prospective study. This paper reports results from the 70 clients who participated in both the retrospective and prospective phases. Of the 34 participants who did not participate in the prospective phase, the most frequent reason for non-participation was difficulty attending the study clinics on a weekly basis due to transport and/or distance problems. Clients who did not agree to participate in the prospective phase were more likely to need an aid for mobilisation (p = 0.017), however there were no differences between these groups for all other demographic, health and ulcer variables.

### Sample characteristics

Participants’ average age was 70 years (range 27–95), 54% were male, 21% required an aid to walk and 72% received an age, unemployment or disability pension. Further information on participants’ demographic, health, co morbidity and ulcer characteristics is shown in Table 1.

**Table 1:** Baseline demographic, health and ulcer characteristics

**Characteristic**	Total^* ^**n = 70**	**Venous n = 32 (46%)**	**Mixed n = 24 (34%)**	**Arterial n = 6 (9%)**	**Diabetic n = 6 (9%)**
***Demographic***					
Age, mean ± SD^†^	67 ± 13.9	64 ± 14.3	71 ± 12.2	75 ± 16.4	67 ± 11.82
Gender:					
female	32 (46%)	18 (55%)	8 (35%)	3 (50%)	3 (50%)
male	38 (54%)	15 (46%)	15 (65%)	3 (50%)	3 (50%)
Lived alone	14 (20%)	6 (18%)	5 (22%)	1 (17%)	2 (33%)
Primary carer	8 (11%)	5 (15%)	3 (13%)	0 (0%)	0 (0%)
Income:					
age or disability pension	44 (64%)	16 (49%)	18 (82%)	6 (100%)	0 (0%)
unemployment benefit	6 (8%)	3 (9%)	0 (0%)	0 (0%)	0 (0%)
employed/self-funded retiree	19 (28%)	12 (36%)	4 (18%)	0 (0%)	1 (17%)
current smoker	10 (16%)	5 (17%)	4 (19%)	1 (17%)	0 (0%)
***Co morbidities/Health***					
Cardiac disease	24 (34%)	5 (15%)	13 (57%)	3 (50%)	2 (33%)
Hypertension	45 (64%)	16 (49%)	18 (78%)	3 (50%)	6 (100%)
Osteoarthritis	30 (43%)	11 (33%)	13 (57%)	2 (33%)	2 (33%)
Rheumatoid disease	8 (11%)	3 (9%)	4 (17%)	0 (0%)	1 (17%)
Other autoimmune disease	7 (10%)	5 (15%)	1 (4%)	1 (17%)	0 (0%)
Diabetes	17 (24%)	2 (6%)	8 (35%)	0 (0%)	6 (100%)
Peripheral arterial disease	16 (23%)	5 (16%)	5 (22%)	4 (67%)	2 (33%)
Past Deep Vein Thrombosis	13 (19%)	9 (27%)	3 (13%)	0 (0%)	1 (17%)
Varicose veins	39 (56%)	21 (64%)	15 (65%)	2 (33%)	1 (17%)
Previous lower limb surgery or trauma	54 (77%)	22 (67%)	21 (91%)	4 (67%)	5 (83%)
History of previous leg ulcers	47 (67%)	24 (73%)	14 (61%)	3 (50%)	5 (83%)
Required an aid to mobilise	15 (21%)	8 (24%)	6 (26%)	0 (0%)	1 (17%)
***Ulcer clinical characteristics on admission***					
Ulcer area (median, range)	2.5 cm^2^ (0.1–45.3)	2.9 cm^2^ (0.8–45.3)	2.5 cm^2^ (0.8 –39.4)	2.35 cm^2^ (0.2–3.8)	1.9 cm^2^ (0.1–9.8)
Ulcer duration (median, range)	22 weeks (2–728)	24 weeks (2–188)	15 weeks (6–728)	13 weeks (10–130)	21 weeks (3–56)
PUSH score (mean ± SD^†^)	9.8 ± 2.9	10.6 ± 2.6	9.9 ± 2.6	8.2 ± 2.9	9 ± 3.5
Lower leg oedema present	55 (79%)	28 (85%)	19 (83%)	4 (67%)	3 (50%)
Venous eczema	12 (17%)	5 (15%)	5 (22%)	0 (0%)	2 (33%)
Clinical signs of wound infection	8 (11%)	4 (12%)	2 (9%)	1 (17%)	1 (17%)

^*^There were two other ulcers which did not fit into the ulcer type categories: one a non-healing wound post-surgery, the other a pressure ulcer on the foot.

^†^SD = Standard Deviation.

### Health service pathways

**Figure 1:**
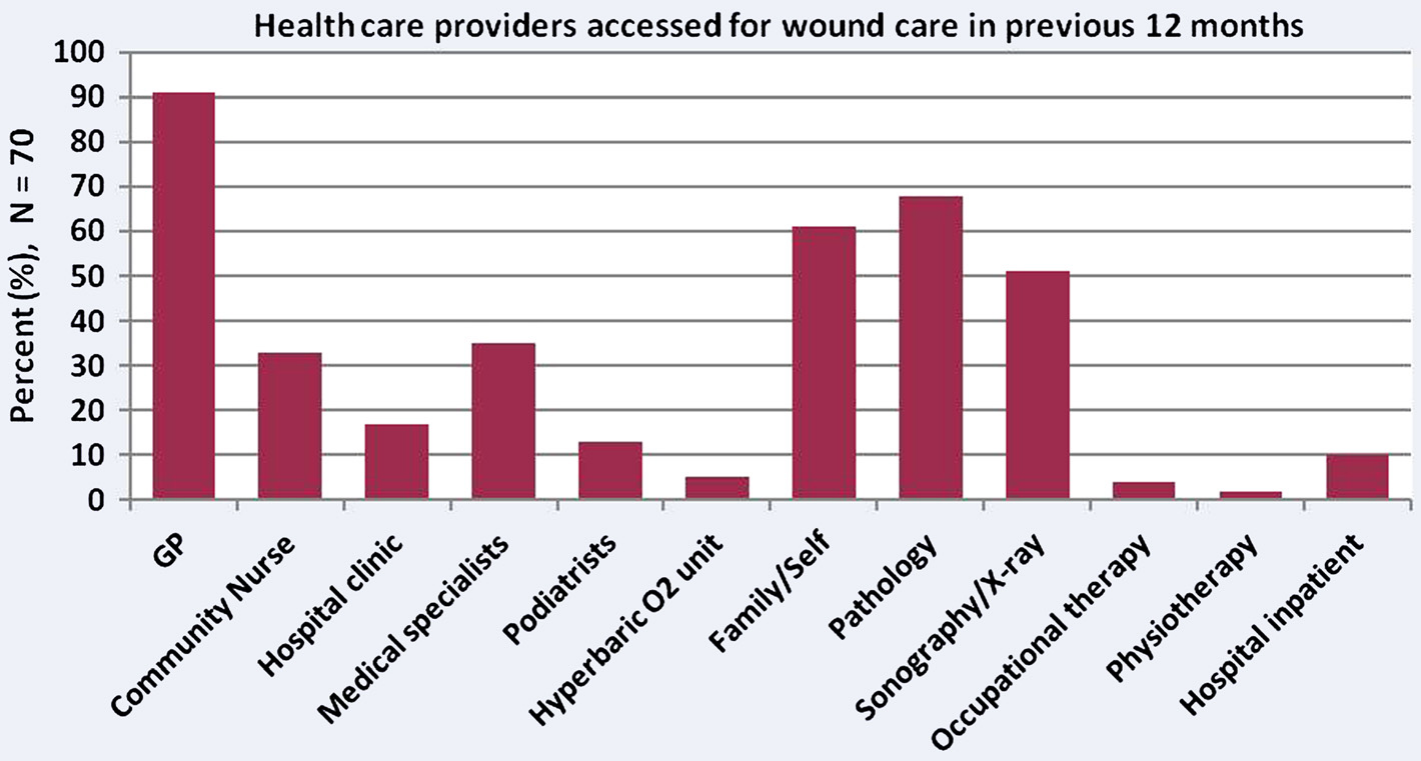
Health service providers in the previous 12 months.

In the 12 months prior to admission, participants had a median of 3 different health specialties and/or organisations involved in providing regular wound care (ranging from 1–8 providers, see Figure 1). They received services from each of these providers on average 2–3 times per week for 17 weeks (range 1–52 weeks).

The most frequent service providers were their local General Practitioners (GPs) (91% were treated by GPs on average twice each week for 16 weeks), and medical specialists (35% were treated by specialists such as vascular surgeons, orthopaedic surgeons, dermatologists, plastic surgeons; on average 1–2 times per week for 16 weeks). Nearly a third of participants (31%) were treated by community nurses on average two or three times each week for 18 weeks; and 77% of participants were referred for pathology, radiology or other specialist medical tests. In addition to health professionals, a significant number of participants self-cared or relied on family members to care for their wounds – 61% self-cared for the ulcer, for an average of 21 weeks, changing dressings three to four times each week.

There were many different combinations of service provider teams, the most frequent being GP care in isolation (42%); GP and allied health professional teams e.g. podiatrist (13%); GP and medical specialist (12%); and GP, medical specialist and community nursing teams (16%). The type of health service provider(s) was significantly related to the participants’ age (p = 0.009) and source of income (p = 0.037). Older participants were less likely to access a GP (p = 0.013) and more likely to receive community nursing care (p = 0.001). Interestingly, males were more likely to access medical specialists than females (p = 0.028). Participants receiving a government pension for income (age, disability or unemployment) were more likely to access community nursing services (p = 0.043), and less likely to self care for their ulcer in comparison to participants who were employed or were self-funded retirees (p = 0.033). The funding system often precludes patients with higher incomes (such as those in employment) from accessing subsidised community nursing services. There were no significant relationships between types of service providers and ulcer duration or aetiology.

In the 24 weeks following admission to the specialist wound clinics, participants had a median of 2 health service organisations and/or specialties involved in their care (range 1–6), on average for one visit each week for 12 weeks. The most frequent service providers were the study wound clinics, where participants received care from either a Medical Practitioner and clinical nurse team member (n = 24, at the hospital outpatient study clinic), or a Nurse Practitioner and clinical nurse team member (n = 46, at the university-based community wound health service); with 100% of participants treated at one of these clinics on average once per week for 12 weeks. Thirty-three percent of participants were treated by GPs on average once per week for 6 weeks; and 37% were treated by community nurses, on average once per week for ten weeks. Nineteen percent of participants saw medical specialists once a week for an average of 5 weeks, and 17% were referred for pathology or other specialist tests. A comparison of the number of each service type provider visits in the six months prior to and six months following admission is shown in Table 2.

**Table 2:** Average number of visits by service provider in the 24 weeks prior to and after admission

**Service provider**	**Mean total no. of visits in 24 weeks pre-admission**	**Mean total no. of visits in 24 weeks post-admission**
General Practitioner	17	1
Community Nurse	7	5
Medical Specialist^*^	4	0.3
Specialist Wound Clinic^**^	0.2	9
Allied Health	0.8	1
Total	29.0	16.3

^*^ includes vascular, dermatology, rehabilitation, geriatric, hyperbaric or plastic surgeon specialists.

^**^ care provided by either a medical practitioner and nurse, or Nurse Practitioner.

### Implementation of evidence based guidelines

A few key evidence based wound management recommendations were chosen as indicators of implementation of evidence based guidelines, i.e. all patients with a lower limb ulcer should have an ABPI or duplex assessment [36-38]; high level compression therapy is the first line of treatment for patients with an uncomplicated venous leg ulcer [36]; and patients at risk or with diabetic foot ulcers require an annual foot examination from a trained professional and should be under the care of a podiatrist as part of a multidisciplinary team [38].

Retrospective study data indicated that levels of implementation of evidence based guidelines were generally low in the twelve months prior to admission to the study clinics. For example, evidence based guidelines recommend all patients with a leg ulcer should have an Ankle-Brachial Pressure Index (ABPI) or duplex ultrasound assessment undertaken every 3–6 months to assist in diagnosis and guidance of treatment [36-38], yet only 31% of participants reported having this undertaken in the previous 12 months. Similarly, venous leg ulcers were the most frequent ulcer type in this sample (n = 32) and compression therapy is the gold standard evidence based treatment [36], however, only 6.3% (2 of 32) of patients with a venous leg ulcer were receiving compression on admission to the study clinics, and a total of 11% had been treated with compression at any time in the previous 12 months. There were only a small number of patients in this sample with a diabetic foot ulcer (n = 6), however, three of the six had not seen a podiatrist or medical specialist in the previous 12 months for a foot examination.

Following admission to a specialist wound clinic, 84% (n = 27) of the participants with venous leg ulcers were treated with compression therapy, 91% of participants had an ABPI assessment undertaken, and 83% of those with a diabetic foot ulcer had a podiatrist and/or high risk foot clinic involved as part of their multidisciplinary wound care team. In the retrospective phase, implementation of these key evidence based recommendations was significantly more likely if participants had specialist health service providers (either vascular/medical specialists or nurse wound care specialists) involved in their care (p = 0.006).

### Wound healing, recurrence and quality of life outcomes

The median ulcer duration on admission to the study was 22 weeks (range 2–728 weeks), with 46% of participants having a wound duration of over six months, and 17% for a year or longer. Sixty-six percent of the participants had a history of previous leg ulcers, 46% of the previous ulcers took more than six months to heal, and 30% had taken over a year to heal.

**Figure 2:**
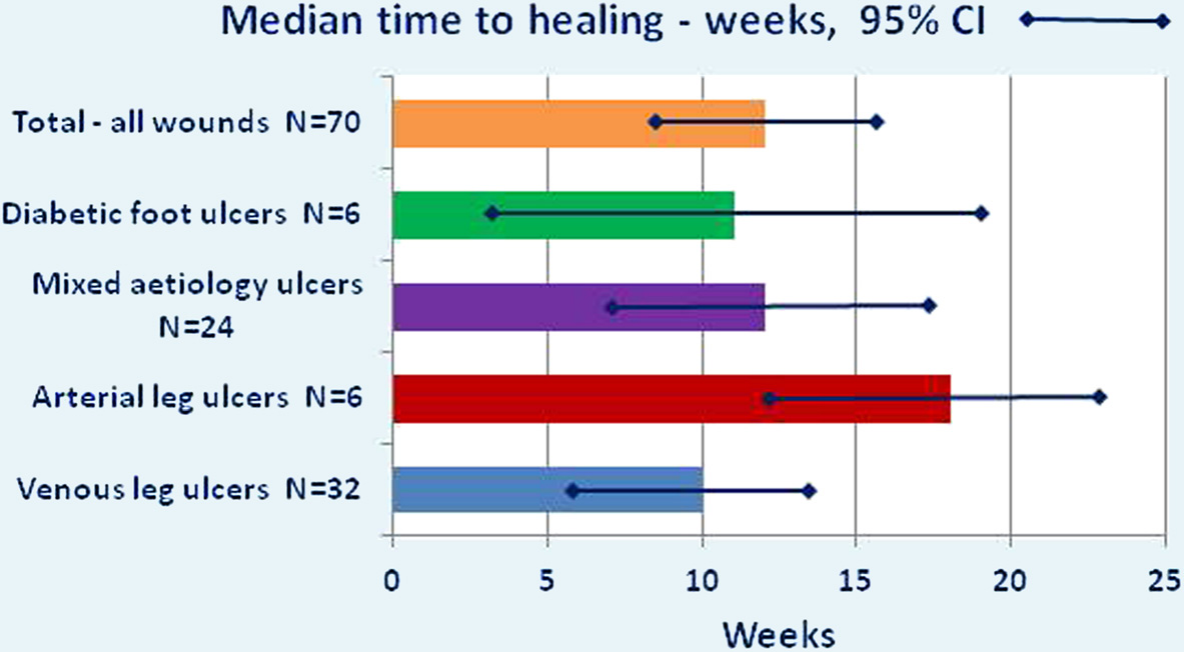
Median time to healing by ulcer type.

Following admission to specialist wound clinics, Kaplan-Meier survival analysis found the median time to healing for the total sample with all wound types was 12 weeks (95% CI 9.3–14.7). Eight participants dropped out of the study before 24 weeks – five due to illnesses and/or hospitalisations unrelated to their leg ulcer, two did not return to the clinics for unknown reasons, and one moved away from the area. Fifty-nine percent (n = 37) of the continuing participants were healed after 12 weeks, and 81% (n = 50) healed after 24 weeks. The median times for healing by each ulcer type are shown in Figure 2. For the largest sub-group in this sample, i.e. participants with venous leg ulcers, healing was significantly associated with implementation of compression therapy (p < 0.001).

Looking at recurrence rates, the most frequent type of ulcer was venous leg ulcers (46%). Sixty-three percent (n = 20) of the participants with venous leg ulcers healed before 12 weeks and 18 of these participants were followed up for another 15 months after healing (2 participants were unable to be contacted and lost to follow-up). In this group, there was a 6% recurrence rate (1 of 18) at three months after healing, and 17% (3 of 18 participants) had recurred by 12 months after healing. The median time to recurrence of venous leg ulcers was 63 weeks (95% CI 52.7–74.1), and for the combined group with all types of ulcers was 56 weeks (95% CI 26.7–64.9). Although this is a small sample, these recurrence rates compare favourably with the venous leg ulcer recurrence rates reported in the literature e.g. three month rates of 25 to 36% [39,40] and 12 month rates ranging from 28 to 68% [23,39,41].

On recruitment to the study, the participants reported lower than average (in comparison to population age norms) health-related quality of life scores: the mean SF-12 Physical Component Summary score was 33.5 (SD 10.5) and mean SF-12 Mental Component Summary score was 46.6 (SD 11.9). Thirty percent of participants scored at mild risk of depression on the Geriatric Depression Scale and another 12% scored at high risk of depression (see Table 3). Participants reported a moderately high pain severity score and 79% of participants required some assistance to perform instrumental activities of daily living, the greatest areas of need being help with housework and shopping. After 24 weeks care at the wound clinics, there was a significant decrease in the number of participants scoring at risk of depression (Chi^2^ 11.9, p = 0.001), significantly decreased pain severity scores (F = 6.08, p = 0.017), and small (non-significant) improvements in health related quality of life. Table 3 provides further detail on the quality of life, depression, pain and functional ability measures.

**Table 3:** Quality of life, pain, depression and functional ability measures

	**Mean ( *SD *) at baseline**	**Mean ( *SD *) at 24 weeks**	***F***	***p***
SF-12 PCS^1^	33.5 (10.5)	34.2 (11.4)	0.314	0.578
SF-12 MCS^1^	46.6 (11.9)	49.9 (10.8)	0.289	0.595
Pain Severity^2^	50.0 (26.4)	34.0 (23.3)	6.08	0.017
IADL Scale^3^	2.50 (1.98)	2.09 (2.02)	11.42	0.002
GDS^4^ scores >4	41.8%	28.9%	*Chi square*11.9	0.001

^1^SF-12 Physical Component Summary (PCS) and Mental Component Summary (MCS) scores, range 0–100, where 50 = population mean and lower scores indicate poorer health related quality of life.

^2^MOS Pain Measures [33], Range 0–100, where higher scores indicate higher levels of pain.

^3^Instrumental Activities of Daily Living Scale [35], range 0 – 7, where 0 = fully independent, and higher scores indicate increased dependence on assistance.

^4^Geriatric Depression Scale [34]: Range 0–15, scores >4 indicate mild risk of depression, scores >10 indicate high risk.

## Discussion

This study highlights the complexity associated with meeting the needs of this population, faced jointly by health service providers, industry, educators and consumers. These include the difficulties accessing health professional and wound care expertise; costs and skills barriers associated with implementation of evidence based care, and the need for evidence on the appropriate health service pathways to facilitate implementation of evidence based wound management and optimal outcomes for patients with chronic leg and foot ulcers.

Poor levels of implementation of evidence based guidelines for leg ulcer care, as found in this sample, have been reported in a number of studies across different countries, along with discussion on possible contributing factors [14,20,21,24]. Costs and inadequate reimbursement associated with evidence based assessment and treatments such as compression therapy have been nominated as a factor hindering best practice in the US [14] and Australia [21], while the difficulties associated with obtaining expertise in skills such as vascular assessment and application of compression bandaging are also known to contribute to delayed implementation of best practice care [14,21]. Many of these skills, such as assessing an ABPI and applying compression, require extensive training and experience to obtain and maintain expertise [14], which may be difficult to organise in busy general primary care settings where the pressures associated with managing high volumes of patients take priority over regular training sessions. However, the absence of a vascular assessment results in inability to safely commence appropriate treatment. The lack of education on wound care as part of health professionals’ routine training is also suggested to contribute to the problem [42]. In studies of health services which have reported successful implementation of evidence based care for patients with leg ulcers, the use of highly trained, specialist nurses or teams to provide care was integral to their success [17,23].

Evidence based guidelines and experts in wound management recommend coordinated specialised leg ulcer services involving care providers at multiple levels, providing continuity and standardisation of care, to obtain optimal outcomes for adults with leg or foot ulcers which fail to show signs of healing within 4–12 weeks [24,43,44]. However, this study found less than half of the participants had been referred to a secondary level of care before admission to the study clinics with an average ulcer duration of 22 weeks. Importantly, results confirmed that involvement of a health care provider with specialist expertise was associated with increased implementation of evidence based guidelines and decreased time to healing. Participants reported a high level of self or family/carer involvement in care for their wounds, often for months at a time – also reported by Nelzen [13] in the Swedish population. In this sample of older adults, three-quarters of whom relied on a government pension, prolonged periods of self-care creates a significant financial and carer burden with regards to costs for dressings, bandages and the time taken from employment and family responsibilities.

Caring for this group of people presents a challenge to health care systems. An increasing trend towards community-based wound care has arisen because of strained resources in the acute care health system and the shift in emphasis from acute sector care to community care. However, coordinated and efficient health service pathways for community-living patients with chronic wounds have not been widely implemented. In agreement with reports by Ghauri et al. [23] and Bulbulia and Poskitt [24], this study confirms that wound care services are frequently provided by an inconsistent mix of primary and specialist health care providers in the community, including GPs and practice nurses, pharmacists, community nursing and personal care personnel, podiatrists, occupational therapists, hospital outpatient clinics, vascular physicians, endocrine/dermatology specialists, and family carers. Chronic wound care consequently has become a hidden, albeit common and costly, problem.

Findings from this study strongly indicate that once patients with leg ulcers begin to receive services from specialised clinics which base their care and treatment on evidence based guidelines, rates of healing significantly increase. Moreover, patients who are cared for in a specialist wound clinic are able to be appropriately assessed according to evidence based guidelines and then able to receive best practice treatments. The results indicate that either current pathways for usual care through GPs and community nursing must adopt evidence based practice for assessment and treatment, or more specialised clinics need to be established. Given the current pressures on GP services and community nursing services it may be challenging to get widespread adoption of evidence based practice in the short term, requiring a major program of education and funding to facilitate penetration of evidence based wound management into these community services. Increasing specialised wound services in key centres would have a two-fold benefit. Firstly, such specialised services could implement immediate changes in assessment and treatment and hence improved healing and reduced costs. Secondly, these specialised services could develop strong collaborations with GPs and community nursing services to become training and education hubs to increase delivery of evidence based practice in other settings. Innovative evidence based hubs would develop broader capacity to improve wound healing and conduct further research on clinical pathways for wound healing.

Patients with chronic leg ulcers report the condition has a significant impact on their general health and normal activities. Restricted mobility associated with pain and multilayered bandages impacts on independence in activities of daily living [45]; with many patients describing social isolation [1,46] and negative psychological impacts such as depression, anxiety and poor body image [47,48]. This study found that in addition to improved healing, other aspects of health improved through the best practice pathways and include reduced pain, improved independence in activities of daily living and improved mental well-being. These improvements result in reduced use of health services which will reduce costs to the health care system and free up health care services to enable greater numbers of patients access to treatment.

### Limitations

Measures of pain, quality of life, functional ability, depression, past treatments and health service use were obtained via interviews and self-report questionnaires and thus have limitations re recall accuracy and response bias. The generalizability of the study results is limited by the descriptive design and the participant sample limited to patients attending the study wound clinics which limits the generalizability of study results.

## Conclusions

There are a number of potential social and economic national benefits to be gained from improving health service coordination for this population: firstly, it can be identified that clients with chronic leg ulcers who are managed in specialist wound clinics have faster healing rates, increased implementation of evidence based care and significantly less use of health services. The outcomes include improved health, well-being and decreased pain for older adults suffering with this condition. A cost effectiveness analysis of these outcomes is currently underway and expected to specifically demonstrate the savings to the health care system arising from these outcomes.

## Competing interests

The authors declare that they have no competing interests.

## Authors’ contributions

HE, KF, MC, NG and MG contributed to study conception and design, CP contributed to data collection and analysis, HE, KF, NG, MG and CP contributed to interpretation of data. All authors were involved in drafting and revising the manuscript and approved the final manuscript.

## Pre-publication history

The pre-publication history for this paper can be accessed here:

http://www.biomedcentral.com/1472-6963/13/86/prepub
